# Analyses of integrated EPID images for on‐treatment quality assurance to account for interfractional variations in volumetric modulated arc therapy

**DOI:** 10.1002/acm2.12805

**Published:** 2020-01-07

**Authors:** Norimasa Matsushita, Mitsuhiro Nakamura, Makoto Sasaki, Shinsuke Yano, Michio Yoshimura, Takashi Mizowaki

**Affiliations:** ^1^ Division of Clinical Radiology Service Kyoto University Hospital Kyoto Japan; ^2^ Human Health Sciences Graduate School of Medicine Kyoto University Kyoto Japan; ^3^ Radiation Oncology and Image‐applied Therapy Graduate School of Medicine Kyoto University Kyoto Japan

**Keywords:** electronic portal imaging device, head and neck cancer, interfraction variation, prostate cancer, volumetric modulated arc therapy

## Abstract

**Purpose:**

To investigate the effects of interfractional variation, such as anatomical changes and setup errors, on dose delivery during treatment for prostate cancer (PC) and head and neck cancer (HNC) by courses of volumetric modulated arc therapy (VMAT) aided by on‐treatment electronic portal imaging device (EPID) images.

**Methods:**

Seven patients with PC and 20 patients with HNC who had received VMAT participated in this study. After obtaining photon fluence at the position of the EPID for each treatment arc from on‐treatment integrated EPID images, we calculated the differences between the fluence for the first fraction and each subsequent fraction for each arc. The passing rates were investigated based on a tolerance level of 3% of the maximum fluence during the treatment courses and the correlations between the passing rates and anatomical changes.

**Results:**

In PC, the median and lowest passing rates were 99.8% and 95.2%, respectively. No correlations between passing rates and interfractional variation were found. In HNC, the median passing rate of all fractions was 93.0%, and the lowest passing rate was 79.6% during the 35th fraction. Spearman’s correlation coefficients between the passing rates and changes in weight or neck volume were − 0.77 and − 0.74, respectively.

**Conclusions:**

Analyses of the on‐treatment EPID images facilitates estimates of the interfractional anatomical variation in HNC patients during VMAT and thus improves assessments of the need for re‐planning or adaptive strategies and the timing thereof.

## 1 INTRODUCTION

Patient‐specific quality assurance (QA) is required when conducting high‐precision radiation therapy, such as intensity‐modulated radiation therapy (IMRT) or volumetric modulated arc therapy (VMAT). This requires a measurement device, such as radiochromic film or an array detector, to verify treatment delivery prior to treatment. The results of such QA measurements are used to inform treatment practitioners about any potential errors derived from the treatment plans and machine conditions that can be detected before treatment. However, pretreatment QA cannot be used to detect interfractional variation, such as setup errors and anatomical changes, which can occur over the course of the treatment. In the case of treatment for prostate cancer (PC), many studies have reported that variation in inter‐ and intrafractional size and position of the prostate and surrounding tissues impacts the doses delivered to the target and the organs at risk (OARs).^1^, ^2^, ^3^ In the case of head and neck cancer (HNC), many researchers have reported that tumor shrinkage and anatomical changes occur during the course of the treatment.^4^, ^5^, ^6^ Barker et al. reported that the parotid glands shift due to weight loss and tumor shrinkage during HNC radiation therapy.^4^ Such anatomical changes cause the doses to the OARs to escalate.^7^

One potential solution to this problem is re‐planning or adaptive radiation therapy, which may help maintain the dose to the target while reducing the doses to the OARs.^8^, ^9^, ^10^, ^11^ However, it is difficult to apply these strategies to all patients due to the large amount of effort required for planning and QA. In addition, the doses must be monitored during treatment courses because we do not know which patients these strategies are appropriate for or the optimal timings.

Recently, on‐treatment QA has been conducted by applying *in vivo* dosimetry methods using an electronic portal image device (EPID).^12^, ^13^, ^14^ Some studies have applied EPID‐based *in vivo* dosimetry to prostate VMAT.^14^, ^15^, ^16^ In those studies, the point dose was estimated based on the photon fluence using a back‐projection algorithm. In other studies, point dose and dose distributions were evaluated using integrated EPID images for various treatment sites,^17^, ^18^, ^19^, ^20^ and gamma analyses were performed throughout VMAT. Kang et al. assessed the relationship between interfractional setup error and integrated EPID images for postmastectomy radiation therapy. Cilla et al. suggested that the discrepancies between planned and delivered photon fluence images might be due to anatomical changes. However, the effects of anatomical changes on photon fluence were not determined in those studies, although investigation of the interfractional anatomical variation during on‐treatment QA would allow the results of the latter to be applied to re‐planning or adaptive treatment strategies.

In this study, we evaluated the integrated EPID images obtained over the course of VMAT, focusing on the anatomical changes in HNC and PC. For this purpose, we used the PerFRACTION software package (SunNuclear Corporation, Melbourne, FL, USA), which is the first reported application of this software for on‐treatment VMAT verification.

## 2 MATERIALS AND METHODS

### 2.1 Patients

This study included 7 patients with PC and 20 patients (19 males and 1 female) with HNC who were given VMAT. The median ages of the PC and HNC patients were 75 (range: 69–82) and 65 (49–74) years, respectively. All PC patients were treated with a full bladder. The patients were in a supine position, five of them were immobilized using BodyFix (Elekta AB, Stockholm, Sweden), and two of them were immobilized in a prone position using a thermoplastic shell (CIVCO Radiotherapy, Orange city, IA, USA). All HNC patients were immobilized using a thermoplastic shell (Klarity Medical Products, Newark, OH, USA). Of the 20 HNC patients, 18 were treated with concurrent chemoradiotherapy. This study was approved by the Institutional Review Board of our university hospital.

### 2.2 Treatment planning

In the PC patients, the prostate and a part of the seminal vesicle were defined and contoured as the clinical target volume (CTV). We added planning target volume (PTV) margins of 8 mm, 7 mm, 6 mm, and 4 mm in the lateral anterior, superior, posterior, and interior directions, respectively. The mean prescribed doses to the PTV were 70–78 Gy. The rectum, bladder, and large and small bowel were defined as OARs. The rectal and bladder walls automatically generated a 4‐mm‐thick structure inward from the rectum and bladder, respectively. All of the plans were optimized using one or two treatment arcs.

All HNC patients were treated using the simultaneous integrated boost (SIB) technique. Three CTVs were defined and contoured. CTV_H_ included the primary tumor or tumor bed, and high‐risk subclinical spread. CTV_I_ included the intermediate‐risk subclinical spread and intermediate‐risk regional lymph node regions, and CTV_L_ included the low‐risk regional lymph node regions. Corresponding PTVs (PTV_H_, PTV_I_, and PTV_L_) were created by adding 5‐mm margin to the CTVs. The spinal cord, brainstem, and parotid glands were contoured as OARs. We defined the planning organ at risk volumes (PRVs) of the brainstem and spinal cord using a 5‐mm margin for each organ (PRV_Stem, PRV_Cord). The prescribed doses for PTV_H_ were 60–70 Gy, depending on the specific characteristics of each patient, and 90% and 80% of the doses prescribed to PTV_H_ were prescribed to PTV_I_ and PTV_L_, respectively. All plans were optimized using two or three treatment arcs.

The dose–volume constraints are summarized in Table 1. Delineations and dose calculations were determined using the Eclipse treatment planning system (ver. 13; Varian Medical Systems, Palo Alto, CA, USA). No patients were re‐planned during the course of treatment.

**Table 1:** Dose–volume constraints for each target and organ

Treatment site	Structure	Dose–volume constraint
Head and neck	PTV_H_	D_50%_ = 100%
		D_98%_ > 93%
		D_2%_ < 105%
	PTV_I_	D_90%_ = 100%
		D_50%_ < 105%
	PTV_L_	D_90%_ = 100%
		D_50%_ < 105%
	CTV_H_	D_95%_ > 100%
	CTV_I_	D_95%_ > 100%
	CTV_L_	D_95%_ > 100%
	GTV	D_95%_ > 100%
	PRV_Stem	D_2cc_ < 54 Gy
	Brainstem	D_max_ < 54 Gy
	PRV_Cord	D_2cc_ < 45 Gy
	Spinal cord	D_max_ < 45 Gy
	Lt. Parotid	V_30Gy_ < 50%
	Rt. parotid	V_30Gy_ < 50%
Prostate	PTV	99% ≤ D_mean_ ≤ 101%
		D_95%_ < 90%
		V_90%_ < 95%
		D_max_ < 110%
	Bladder wall	V_40Gy_ < 65%
		V_70Gy_ < 35%
	Rectal wall	V_40Gy_ < 65%
		V_60Gy_ < 35%
		V_70Gy_ < 25%
		V_78Gy_ < 1%

Abbreviations: Dmax, maximum dose; D_mean_, mean dose; D_xx_, dose covering xx% volume; Lt., left; PRV, planning organ at risk volume; Rt., right; V_yyGy_, volume receiving yy Gy.

### 2.3 Treatment delivery

The treatments were delivered with a TrueBeam or TrueBeam STx (Varian Medical Systems) equipped with an amorphous silicon EPID (aS‐1200) with 16‐bit quantization levels, a matrix size of 1,190 × 1,190 pixels, and pixel size of 0.336 mm. In PC patients, cone‐beam computed tomography (CBCT) images were obtained using the onboard imager (OBI) system (Varian Medical Systems) to correct the initial setup positions based on localization of the prostate. The initial setup positions of the HNC patients were corrected based on the vertebral bone anatomy using ExacTrac (BrainLAB, Heimstetten, Germany) or the OBI system. On‐treatment integrated EPID images were obtained as the photon fluence at the position of the EPID for each treatment arc.

### 2.4 Analyses of the photon fluence

**Figure 1:**
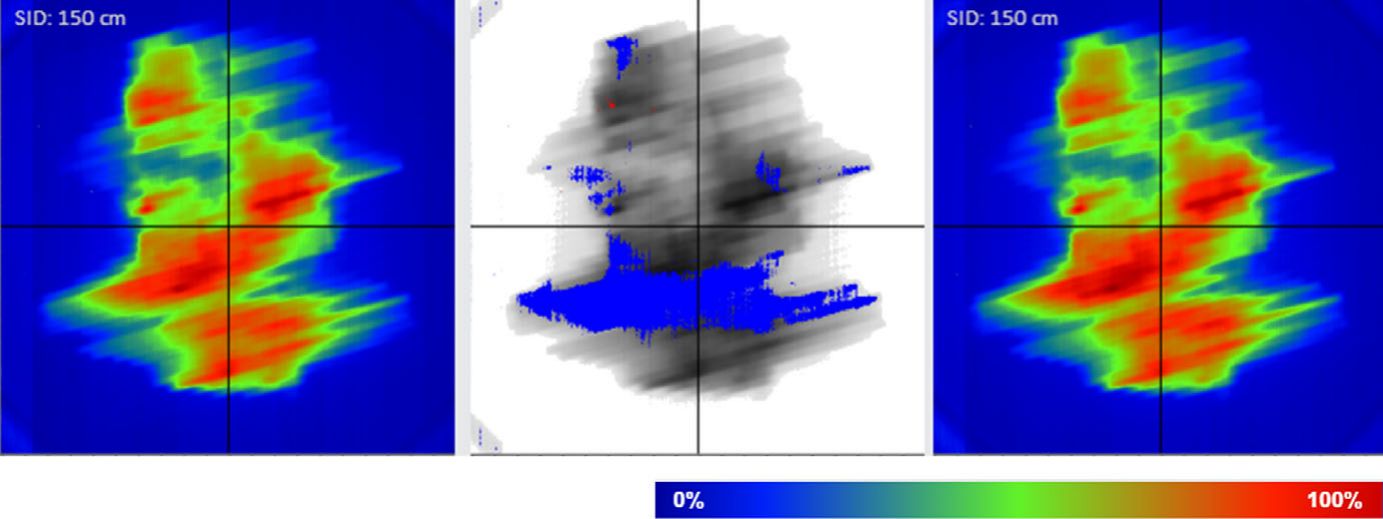
Analysis of photon fluence using the PerFRACTION software. Left: the photon fluence of subsequent fractions; right: the photon fluence of the reference fraction. The middle image shows the difference between the left and right images. The blue area indicates the failed points.

Figure 1 shows the analysis of the integrated photon fluence. The differences between the fluence in the first fraction and those in subsequent fractions were calculated for each arc. When the integrated EPID images could not be acquired in the first fraction due to mechanical or human error, the fraction in which the integrated EPID image could be first acquired was chosen as the reference fraction instead of the first fraction. There were 13 and 48 arcs for PC and HNC cases, respectively. The passing rate was then calculated based on a tolerance level of 3% of the maximum fluence. These analyses were performed using the PerFRACTION software package for areas exposed to> 10% of the maximum fluence.

### 2.5 Correlation between anatomical changes and the passing rate

In PC cases, we calculated the rectal and bladder volumes during the treatment by contouring them on CBCT images. The rectal volume was defined as the volume extended by 4 cm in both the superior and inferior directions from the slice where an isocenter was located. The changes in the bladder and rectal volumes (∆*V*) were calculated as follows:

(1) $$\Delta V=\frac{V_{n}-V_{1st}}{V_{1st}}\times 100\left(\%\right),$$

where *V_n_* is the volume in the *n_th_* fraction, and *V*_1_ *_st_* is the volume in the first fraction.

In HNC cases, we investigated the relationship between the passing rates and weight as well as changes in the neck volume after the first fraction. We included 13 patients who had measured their weight during their hospitalization in this part of the study. Their weight was not measured every day. Additionally, the neck volumes of 10 patients were evaluated using CBCT images obtained at the *k* ^th^ (*k *= 1, 5, 10, 15, 20, 25, and 30) and last fraction. The neck was defined as extending from the C1 to the C4 vertebra. The entire enclosed volume was identified within the field of view (FOV). A change in body weight or neck volume (∆*X*) was defined as follows:

(2) $$\Delta X=\left|\frac{X_{n}-X_{1st}}{X_{1st}}\right|\times 100\left(\%\right),$$

where $X_{n}$ is the body weight or neck volume in the *n_th_* fraction, and *X*_1_ *_st_* is the corresponding value in the first fraction.

The relationship between the passing rate and anatomical changes, namely, the change in body weight or neck volume, was evaluated using Spearman’s correlation coefficient.

## 3 RESULTS

### 3.1 Prostate cancer cases

The passing rates for the PC patients were stable and higher than 95% for all patients. The median and lowest passing rates were 99.8% and 95.2%, respectively, throughout the course of the treatment. The passing rates were higher than 99.0% for 90.5% of arcs.

**Figure 2:**
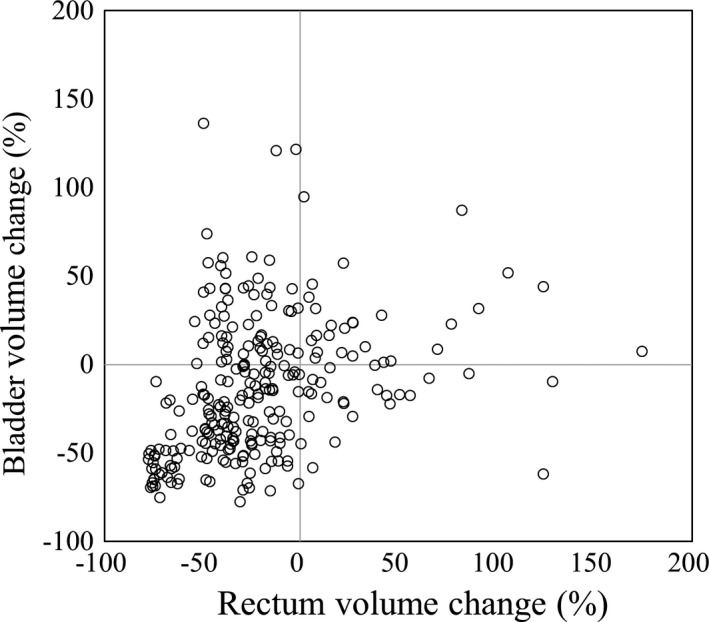
Rectal and bladder volumes relative to their values in the first fraction. The rectal and bladder volumes were smaller in subsequent fractions than in the first fraction in 80.0% and 66.3% of the fractions, respectively. The maximum changes in the volumes of the rectum and bladder were 174.7% and 136.1%, respectively.

Figure 2 shows the variation in the rectal and bladder volumes over the course of the treatment. The rectal and bladder volumes were smaller in subsequent fractions than in the first fraction in 80.0% and 66.3% of the fractions, respectively. The maximum changes in the volumes of the rectum and bladder were 174.7% and 136.1%, respectively. Even in those fractions, the passing rates were higher than 99.8%. We did not find any correlations between the passing rate and interfractional variation.

### 3.2 Head and neck cancer cases

**Figure 3:**
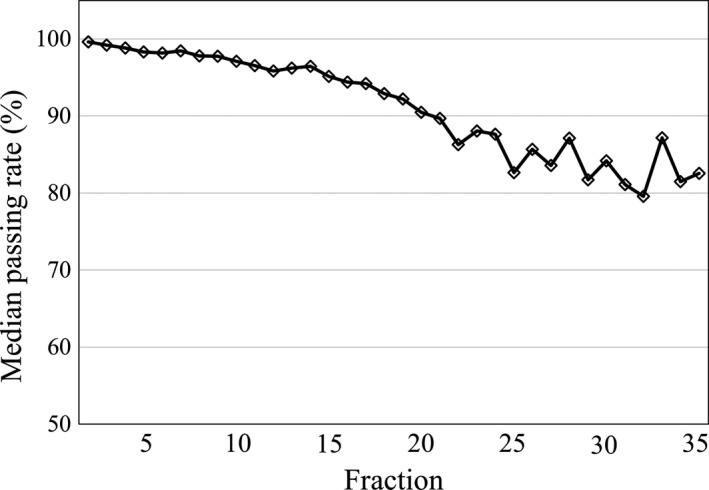
The median passing rates over the course of treatment for head and neck cancer (HNC). The passing rates generally decreased as treatment progressed. The median passing rate of all fractions was 93.0%, and the lowest passing rate was 79.6% during the 35th fraction.

**Figure 4:**
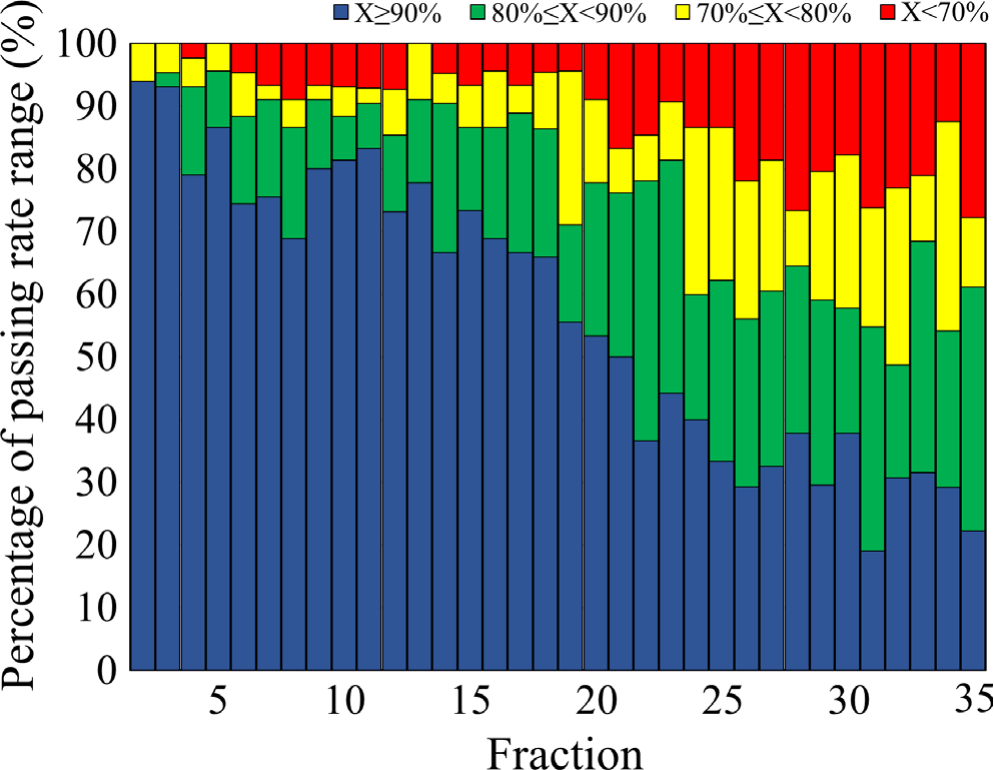
Distribution of the HNC patients among the passing rate ranges. The passing rate ranges were as follows: blue: >90%, green: 80–90%, yellow: 70–80%, red: <70%. The percentage of passing rates> 90% decreased as treatment progressed, and the percentage of passing rates < 70% increased after the 20th fraction.

**Figure 5:**
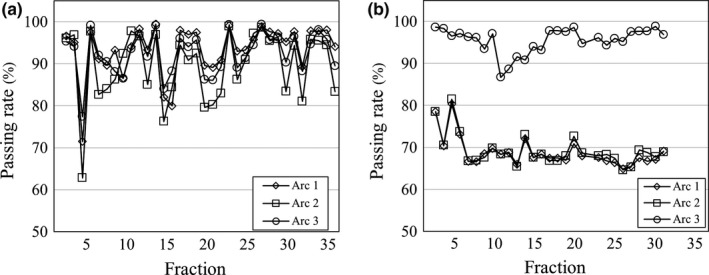
The passing rates in two HNC patients in which a decrease in the early fractions occurred independently of any anatomical change. Both plans consisted of three treatment arcs. The passing rate was < 80% until the fifth fraction, but large anatomical changes did not occur in either of the HNC patients, whose neck volume changes in the fifth fraction were (a) 2.4% and (b) 0.4%, respectively.

Figure 3 shows the median passing rates of each fraction during treatments for HNC cases. In contrast to PC cases, the passing rates generally decreased as the treatment progressed. The median passing rate of all fractions was 93.0%, and the lowest passing rate was 79.6% during the 35th fraction. Figure 4 shows the distributions of the passing rates in each fraction. We can see that the percentage of passing rates over 90% decreased as the treatment progressed, and the percentage of passing rates under 70% increased after the 20th fraction. The overall percentages of passing rates> 90%, between 80 and 90%, between 70 and 80%, and < 70% were 57.5%, 20.5%, 11.5%, and 10.5%, respectively. A lower passing rate was found in some of the early fractions; for example, the percentage < 90% was 13.3% in the fifth fraction. Figure 5 shows two cases in which the passing rate decreased in the early fractions. In the case shown in [Fig. 5(a)], the variation in the passing rate was large, whereas in the case shown in [Fig. 5(b)], the passing rate of arc 1 and arc 2 was < 80% throughout treatment, with the exception of the fourth fraction.

**Figure 6:**
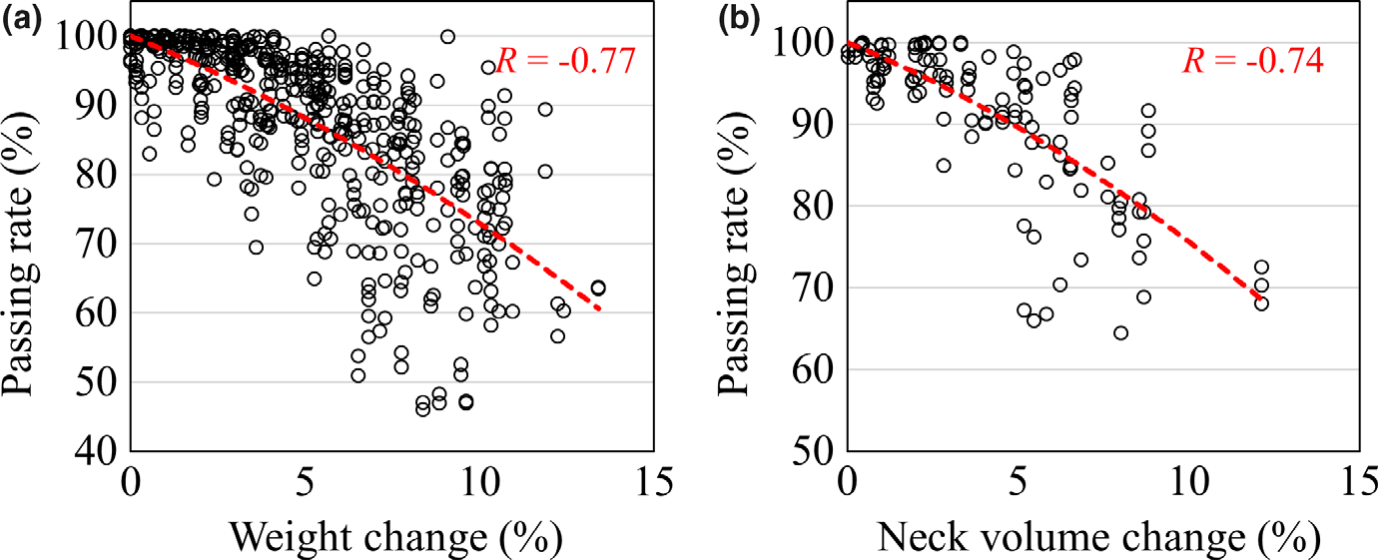
Relationships between the passing rates and changes in (a) weight and (b) neck volumes. The red curves represent the quadratic approximations, with Spearman’s correlation coefficients denoted as *R*. Spearman’s coefficients for the correlations between the passing rates and changes in weight or neck volume were − 0.77 and − 0.74, respectively.

Figure 6 shows the relationship between passing rate and weight loss, which were highly correlated (Spearman’s correlation coefficient = −0.77, *P* < 0.01). We observed neck volume loss in 9 of 10 cases. The maximum neck volume loss was 12.3%. As shown in the figure, the passing rate was closely correlated with changes in neck volume (Spearman’s correlation coefficient = −0.74, *P* < 0.01).

## 4 DISCUSSION

In this study, the interfractional variation was estimated using on‐treatment EPID images and a passing rate based on a tolerance level of 3% of the maximum fluence. An initial gamma analysis lacked the sensitivity needed for the detection of anatomical changes; therefore, an intensity difference analysis was used because of its greater sensitivity in recognizing interfractional variations.

The passing rates of PC cases were higher than 95% throughout the course of the treatment, regardless of interfractional variation in the volumes of the bladder and rectum. Although the rectal and bladder volumes changed over the course of PC treatment, the passing rates were> 95%. The photon fluence was affected by the water equivalent beam path length in patients, and in turn, this was affected by changes in rectal volume due to gas conditions during prostate treatment. We did not consider the variation in the water equivalent path length due to rectal gas to have caused the 3% differences between the photon fluences in VMAT for PC cases.

Unlike in PC, the passing rates in HNC generally decreased as treatment progressed. Cilla et al. conducted a study on ongoing patient QA using integrated EPID images in HNC VMAT cases.^20^ In their investigation of the gamma passing rates of integrated EPID images, they determined a gamma passing rate (3%/3 mm) of 92.9%, which suggested that the discrepancies between the photon fluences of the first fraction and subsequent fractions were caused by patient positioning errors and anatomical variation. We demonstrated that weight loss (*R* = −0.77) and changes in neck volume (*R* = −0.74) were strongly correlated with the interfractional variance of on‐treatment EPID images. The largest factor contributing to the passing rates in HNC cases was the reduction in the water equivalent path length caused by weight and neck volume loss.

In this study, the contribution of patient positioning error to the passing rate could not be investigated. As shown in Fig. 5, the passing rate was < 80% until the fifth fraction, but it did not lead to large anatomical changes in the two HNC patients whose neck volume changes in the fifth fraction were 2.4% and 0.4%, respectively. This decrease in the passing rate may have been due to patient positioning. Figure 5(b) shows that the passing rate of arc 3 was higher than those of arcs 1 and 2. Whereas the beams of the latter arcs likely passed through the shoulder region, the beam of arc 3 passed only through the neck region. Tachibana et al. reported that shoulder deformation in the superior–inferior and anterior–posterior directions in HNC patients affected a water equivalent path length in VMAT.^21^ In all fractions, the patient positions, including the shoulder positions, were set using laser and skin tattoos and then corrected using ExacTrac or the OBI system. However, the shoulder positions could not be entirely corrected or evaluated under image guidance, because the clavicle or humeral head was not included within the FOV of the ExacTrac system. The residual setup error was not recorded. In addition, intrafractional motion may have occurred, because the shoulder could not be immobilized with the thermoplastic shell. Zhuang et al. reported that a setup error as small as 1 mm and 0.5° could be detected using an anthropomorphic phantom with the PerFRACTION software.^22^ As described above, the lack of a setup error evaluation was a limitation of our study. In addition, the impact of a change in the EPID calibration on the passing rate could not be assessed, because the consistency of the EPID response was not checked during this study. However, we consider any impact of a change in the EPID calibration to be small. Because the passing rate in the PC cases was stable, the EPID image was obtained during the same period as that in the HNC cases.

**Figure 7:**
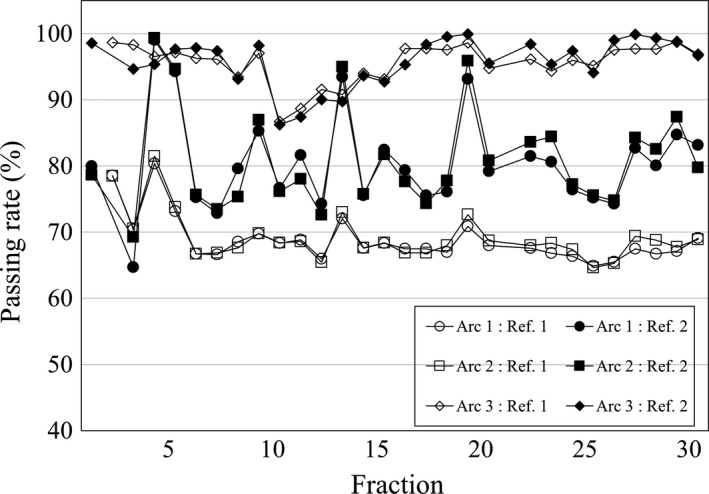
Translation of the passing rate when a reference fraction was changed from the first fraction to the second fraction. The passing rates of arcs 1 and 2 were higher when the second rather than the first fraction served as the reference fraction.

The implication of this kind of verification system for clinical situations is that the patient dose must be delivered very carefully in the first fraction, because this fluence is typically used as the reference for the subsequent fractions. If the passing rates largely decrease after beginning treatment despite the clinically acceptable results from the pretreatment QA, the possibility of large anatomical changes, patient setup errors, or other treatment delivery issues, such as machine output error, should be considered. In such cases, correct delivery of the doses should be determined not only in that fraction but also in the first fraction. Figure 7 shows the higher passing rate when the fluence in the second rather than the first fraction was selected as the reference. The decrease in the passing rate may have been due to interfractional variation of the shoulder position, especially in the first fraction.

The passing rates in HNC cases will be used as the primary criteria to evaluate and select patients who need re‐planning or adaptive radiation therapy, as well as determine the optimal re‐planning schedule. According to a previous report, one major advantage of using adaptive strategies is to prevent doses to the parotid glands.^23^ Some studies have reported that body weight loss is related to shrinkage of these glands, and that they shrink more as the mean dose to the gland increases.^2^, ^24^, ^25^ Capelle et al. reported that the reduction in the diameter of the neck caused the mean dose to the parotid gland to increase.^24^ In this study, we estimated these anatomical changes by analyzing on‐treatment EPID images. Using this method may make it possible to identify unexpected escalations in doses to parotid glands. We will need to identify the appropriate action levels for differences in doses so that we can determine the optimal re‐planning schedules.

## 5 CONCLUSION

The passing rates were stable for the PC cases but decreased substantially throughout the treatment course for most of the HNC cases, in which they were highly correlated with anatomical changes. Analyzing on‐treatment EPID images enabled us to estimate the interfractional anatomical variation in HNC patients during VMAT, helping us to assess the need for and timing of re‐planning or adaptive strategies.

## CONFLICT OF INTEREST

No author has any conflict of interest to declare.
